# Patient reported outcomes of symptoms and quality of life among cancer patients treated with palliative pelvic radiation: a pilot study

**DOI:** 10.1186/1756-0500-4-252

**Published:** 2011-07-21

**Authors:** Marte G Cameron, Christian Kersten, Rene van Helvoirt, Gudrun Rohde, Sophie D Fosså, Ingvild Vistad

**Affiliations:** 1 Center for Cancer Treatment, Sørlandet Hospital Trust, Service Box 416, 4604 Kristiansand, Norway; 2 Faculty of Health and Sport Sciences, University of Agder; Research Unit, Sørlandet Hospital Trust, Kristiansand, Norway; 3 Oslo University Hospital, Norwegian Radium Hospital; University of Oslo, Oslo, Norway; 4 Department of Gynaecology, Sørlandet Hospital Trust, Kristiansand, Norway

## Abstract

**Background:**

There is limited high-quality research investigating the efficacy of palliative radiation (PPR) with regard to symptoms and quality of life (QOL) among cancer patients with pelvic soft tissue tumors. As a result, clinicians are left with mainly retrospective studies, without reliable data on which to base treatment decisions. As a first step of a subsequent analysis of PPR's efficacy, we aimed to determine whether it is feasible to prospectively measure symptoms and QOL among patients treated with PPR. A secondary aim was to explore patients' willingness to answer existential questions in the setting of palliative pelvic radiation.

**Methods:**

Patients referred for palliative radiation of soft-tissue pelvic tumors were invited to enter the study. Symptoms were scored by study physicians and QOL was assessed by the EORTC QLQ C-30 questionnaire and site specific modules (PR25, CR38 or BL24) prior to start of radiation and 6 and 12 weeks after its completion. In addition, patients answered existential questions at each of the study visits. A radiation therapist was available to participants in order to answer their questions and ensure that questionnaires were completed.

**Findings:**

Five female and 17 male patients with prostate cancer (14), colorectal cancer (5) and bladder cancer (3) were included in the study. The median age of the participants was 75 years (range 62-90). Twenty patients were still in the study at the 6-week follow-up and 18 patients at the 12-week follow-up. Twenty-one patients had valid responses within all the EORTC QLQ C-30 scales at baseline, 20/20 at the 6-week follow-up and at the 12-week follow-up 17/18 patients still in the study had valid responses within all scales. This level of response was similar in the site-specific modules and among the existential questions.

**Discussion:**

Among patients with prostate, colorectal and bladder cancer, compliance to questionnaires assessing symptoms, QOL and existential questions 6 and 12 weeks after PPR is sufficient to enable evaluation in a larger and more homogeneous patient group in order to reach clinically valid conclusions as to the efficacy of PPR.

## Background

The incidences of prostate, colorectal and bladder cancers continue to rise in many western societies [1] as well as in many developing countries as they adopt a more "western" lifestyle [2]. Steadily increasing life-expectancy contributes to increased incidence [3] and with advancements in systemic treatments such as hormonal manipulation, biological agents and chemotherapy, patients can potentially live for many months and even years with advanced stages of malignancy. Palliative pelvic radiation (PPR) is a treatment option with a long clinical tradition in cases of symptomatic pelvic tumors [4-6].

In PPR, there exists a fine balance between ameliorating cancer symptoms versus the potential drawbacks of treatment toxicity and complications, as well as valuable time spent ("lost") in treatment. Radiation oncologists use PPR to treat pain, bleeding, and obstruction, in an effort to indirectly enhance patients' quality of life (QOL) [7]. Physician assessment of symptoms and patient well-being often falls short [8] and ultimately, it is the patients' subjective experience of symptom burden, treatment-related side effects and quality of life that are the important and clinically valid endpoints in palliation. There is, however, limited evidence-based information to support the efficacy of PPR with regard to symptoms and QOL of patients with bladder cancer [9,10] and even less so in cases of prostate [11], and colorectal [12] cancers.

Potential areas of practical and ethical conflict in the investigation of palliative treatments include: (a) defining the patient group, (b) inclusion and follow-up of terminally ill patients in a research protocol, and (c) addressing the effects of confounding treatments [13]. Consequently, palliative treatment regimens are often based on local tradition and clinical anecdotes, without hard scientific evidence.

To the best of our knowledge, there exist no published prospective evaluations of PPR among patients with prostate, and colorectal cancer, and only one randomized trial among patients with bladder cancer, that adequately describe its effects on symptoms and QOL. In order to clarify the indication for and dosage of this common procedure, reliable documentation of its effects is necessary. Due to the challenges inherent to this type of research, a pilot study was regarded as a natural first step in this process. The purpose of this study was to determine whether it is feasible to prospectively measure symptoms and QOL among patients treated with PPR. A secondary aim was to explore patients' willingness to answer existential questions in the setting of palliative pelvic radiation.

## Methods

### Patients

All patients referred to the Center for Cancer Treatment, Sørlandet Hospital Trust, Kristiansand for fractionated palliative radiation of soft-tissue pelvic tumors were screened for eligibility. Eligibility criteria were as follows: age ≥ 18 years, histologically or cytologically proven colorectal (CRC), bladder (BC), or prostate cancer (PC), planned palliative fractionated radiotherapy of soft tissues (not skeletal metastases), life expectancy > 3 months, ability to understand spoken and written Norwegian, no significant cognitive impairment, no treatment with investigational therapy and signed informed consent.

In our institution, fractionated pelvic radiotherapy is given to patients with Eastern Cooperative Oncology Group (ECOG) functional status [14] two or better. ECOG functional status three or worse was therefore an indirect exclusion criterion. Due to the exploratory nature of the feasibility study, concomitant treatment with other anti-tumor therapies (chemotherapy, hormonal manipulation, etc.) was not an exclusion criterion.

### Radiation treatment

Fractionation schemes were determined by the treating radiation oncologist prior to referral to the study. Two to four radiation fields with six or 15 megavoltage photon beam radiation were used. Treatment fields were planned based on computed tomography of the pelvis and the target volumes consisted of gross tumor with 1-2 cm margins.

### Measurements/evaluation

There were three study visits. The baseline evaluation took place just prior to radiation, and follow-ups were done six and twelve-weeks after completion of radiotherapy. At each visit, the study physician completed a prospective evaluation of symptoms, functional status, medications and complications. Participants completed questionnaires while in the treatment center, assisted by a radiation therapist when necessary. Blood tests, consisting of hematology, liver and renal function, electrolytes, and tumor markers were taken as pre-radiation routine.

QOL was assessed by the European Organization for the Research and Treatment of Cancer Quality of Life Questionnaire C-30 (EORTC QLQ C-30, v.3.0) core questionnaire, developed and validated for use among cancer patients world-wide [15]. It covers aspects of QOL considered to be relevant to most cancer patients, and includes five functional scales (physical, role, emotional, cognitive and social), three symptom scales (fatigue, pain and nausea and vomiting), a global QOL scale as well as five symptoms common among cancer patients (dyspnoea, anorexia, insomnia, constipation and diarrhea) and perceived financial impact of the disease and treatment. This questionnaire has been validated for use among Norwegian patients with heterogeneous cancer diagnoses [16] and among those receiving palliative radiation [17]. In addition, patients filled out site specific modules, depending on their diagnosis (PR25 = Prostate cancer module [18], CR38 = Colorectal cancer module [19] or BL24 = Bladder cancer module), in order to cover additional aspects of QOL considered relevant to these specific cancer types.

At each of the three study visits, patients also answered a seven-item module of questions regarding existential issues and life outlook, extracted from the 81-item Impact of Cancer (IOC) Instrument [20]. These questions can be found in Table five.

### Analysis

Descriptive statistics were used to summarize patient accrual, survey completion, survival and withdrawals from the study.

### Ethics

Participants were given written and oral information about their planned palliative radiation treatment and about the pilot study by an oncologist. All participants signed an informed consent form. Approval for the study was granted by the Regional Ethics Committee, the Norwegian Social Science Data Services and the Hospital Research Board.

## Findings

The study screened 26 and enrolled 22 patients between March 2008 and April 2009 (table 1). Reasons for non-enrollment were patient choice (belief that the study procedure and questionnaires were too demanding) in three cases and cancellation of planned radiotherapy due to clinical deterioration and progressive disease in one case.

**Table 1:** Characteristics of included patients (N = 22)

Age, years	
**Median**	**75**
**Range**	**62-90**

**Sex**	

**Male**	**17**
**Female**	**5**

**Diagnosis**	

**Prostate cancer**	**14**
**Colorectal cancer**	**5**
**Bladder cancer**	**3**

**Baseline ECOG performance status**	

**0**	**3**
**1**	**14**
**2**	**5**

**Radiation schedules**	

**2 Gy × 25 = 50 Gy**	**7**(6 PC, 1 CRC, 1 BC*)
**3 Gy × 10 = 30 Gy**	**6**(3 PC, 2 CRC, 1 BC)
**2 Gy × 30 = 60 Gy**	**4**(4 PC)
**3 Gy × 13 = 39 Gy**	**2**(1 CRC, 1 BC)
**2 Gy × 20 = 40 Gy**	**1**(1 PC)
**4 Gy × 5 = 20 Gy**	**1**(1 CRC)

**Survival (in months) from last radiation treatment**	

**3 month survival**	**91%**
**6 month survival**	**73%**
**1 year survival**	**68%**
**2 year survival**	**36%**

*One patient with bladder cancer did not complete the planned radiotherapy regimen (completed 13 fractions of the planned 2Gy × 25) due to general fatigue and a wish to be discharged from the hospital. The remaining 21 patients completed their prescribed treatments.

Eight patients were still alive 18 months after the pilot study was closed. All but one patient survived for the duration of the study (duration of radiation treatment plus 12 weeks follow-up). Three patients did not complete the study due to clinical deterioration and one patient moved away from the region prior to the 6 week follow-up.

Radiotherapists assisted patients as-needed and encouraged them to complete the questionnaires independently. The amount of time used per patient ranged from zero to 30 minutes. The primary reasons for radiotherapist assistance were difficulty reading questions and difficulty with written responses. In addition, there were occasional issues of question clarification and reminders to fill out the forms in their entirety (table 2).

**Table 2:** EORTC QLQ completion rates

Study contact	Number of completed C30 and site-specific questionnaires/eligible patients	Diagnoses of patients who filled out the questionnaires/eligible patients	Overall response rates
Baseline	21/22	14/14 PC 4/5 CRC 3/3 BC	95%

6 week follow-up	20/22	13/14 PC 5/5 CRC 2/3 BC	91%

12 week follow-up	17/22	11/14 PC 4/5 CRC 2/3 BC	77%

Questions regarding sexuality were answered by 20 patients (91%) at baseline, 18 (82%) at the 6-week follow-up and 13 (59%) at the 12-week follow-up. These were the most frequent single-item omissions.

Pre-treatment responses to EORTC QLQ-C30 (table 3) and existential questions (table 4) are reported in order to give an indication of the baseline symptom burden and general health of our cohort.

**Table 3:** Baseline responses to EORTC QLQ-C30

				Not at all (n)	A little (n)	Quite a bit (n)	Very much (n)
1. Do you have any trouble doing strenuous activities like carrying a heavy shopping bag or a suitcase?				6	8	5	2
2. Do you have any trouble taking a long walk?				7	4	5	5
3. Do you have any trouble taking a short walk?				14	3	2	2
4. Do you need to stay in bed or a chair during the day?				5	6	7	3
5. Do you need help with eating, dressing, washing yourself or using the toilet?				21	0	0	0
***During the past week:***							
6. Were you limited in doing either your work or other daily activities?				4	9	4	4
7. Were you limited in pursuing your hobbies or other leisure time activities?				5	6	4	5
8. Were you short of breath?				14	1	6	0
9. Have you had pain?				6	5	8	2
10. Did you need to rest?				1	9	8	3
11. Have you had trouble sleeping?				11	5	3	2
12. Have you felt weak?				5	8	5	3
13. Have you lacked appetite?				10	6	3	2
14. Have you felt nauseated?				15	4	2	0
15. Have you vomited?				17	3	0	0
16. Have you been constipated?				8	8	2	3
17. Have you had diarrhea?				14	4	2	1
18. Were you tired?				3	9	6	3
19. Did pain interfere with your daily activities?				7	5	5	4
20. Have you had difficulty in concentrating on things, like reading a newspaper or watching television?				18	2	1	0
21. Did you feel tense?				14	5	1	1
22. Did you worry?				11	8	1	1
23. Did you feel irritable?				13	5	3	0
24. Did you feel depressed?				11	7	2	0
25. Have you had difficulty remembering things?				13	5	3	0
26. Has your physical condition or medical treatment interfered with your family life?				10	4	6	1
27. Has your physical condition or medical treatment interfered with your social activities?				6	7	6	2
28. Has your physical condition or medical treatment caused you financial difficulties?				20	1	0	0
	**Very** ** poor**						**Excell-** **ent**
	**1**	**2**	**3**	**4**	**5**	**6**	**7**
29. How would you rate your overall health during the past week? (n)	0	1	7	5	2	4	2
30. How would you rate your overall quality of life during the past week? (n)	0	1	4	5	4	5	2

As answered at baseline by 21 of the 22 included patients. There were three single-item omissions (questions 7, 15 and 24) among these 21 responders.

**Table 4:** Seven Existential questions taken from the IOC Instrument

	Completely agree (n)	Agree (n)	Neutral (n)	Disagree (n)	Completely disagree (n)
***Positive Outlook***					

Having had cancer has made me realize that time is precious.	8	7	4	0	2

Having had cancer has strengthened my religious faith or my sense of spirituality.	9	0	8	2	2

I learned something about life because of having had cancer.	3	10	6	0	2

***Negative Outlook***					

Having had cancer makes me feel unsure about my future.	6	5	6	1	3

I worry about my future.	3	5	4	3	6

I am afraid to die.	1	3	4	6	7

I feel like time in my life is running out.	3	5	2	9	2

As answered at baseline by 21 of the 22 included patients. There were no single item omissions.

21/22 patients answered the IOC questions about existential matters at baseline. At the six week follow-up 19 patients answered the existential questions fully and at the 12-week follow-up 16 patients answered the existential questions fully.

## Discussion

The findings of the present pilot study show that it is feasible, within a research project, to prospectively evaluate symptoms, QOL and existential issues among patients undergoing PPR for locally advanced prostate, colorectal and bladder cancers.

Patient accrual in this pilot study was good, with 85% of potential candidates included, despite a rather demanding protocol, with over two hundred questionnaire items per participant.

Study withdrawal was the largest contributor to the decline in response rates between baseline and the six and 12-week follow-ups. Reasons for study withdrawal depended on patients' declining general health. This is to be expected in a population with such advanced malignancy and relatively limited life-expectancy [22].

For the patients that remained in the study for its duration, however, completion of questionnaires did not appear to be too rigorous and as seen in previous reports, it was the questions related to sexuality that were most commonly omitted by patients filling out the EORTC questionnaires [23]. In our small cohort, patients who were physically able to come to the follow-up appointments all filled out the required questionnaires sufficiently and reported that they enjoyed participating, despite the fact that the questionnaire procedure required roughly thirty minutes of additional time spent at each of the three study visits. The fact that the radiation therapist ensured that the forms were complete prior to patients leaving the treatment center is likely to have improved questionnaire response rates [24].

This feasibility study used clinically acceptable methods, while exploring the question of QOL using validated research tools (BL24 was the only module not finally validated). An overly ambitious protocol can hamper accrual, questionnaire response rates, and study completion, particularly in a palliative population. The EORTC QLQ questionnaires were chosen because of their comprehensiveness, ease of use, and the high levels of reliability and validity they have demonstrated in two decades of international research [25]. We chose the EORTC QLQ-C30 and its corresponding diagnosis-specific modules rather than the EORTC palliative module (QLQ-C15-PAL) because of the more comprehensive symptom data that could be gathered using the diagnosis-specific modules.

The use of selected existential questions taken from the IOC instrument is a limitation of this pilot study. Psychometric tests of these items were not carried out on our small cohort and as far as we know, these questions have not been tested for validity or reliability among patients with advanced cancer. The complete IOC questionnaire, which is a larger and more complex instrument, has been psychometrically tested among long-term cancer survivors [27]. Fundamental differences between the context of palliative treatment and the context of long-term cancer survivorship are likely to impact on the responses to existential questions, thereby limiting our ability to interpret these findings.

This pilot study did not seek to evaluate the effects of the PPR but to test the feasibility of such an evaluation. With a hypothetical primary endpoint of QOL at 12 weeks post-radiation, 17 patients (77%) would have been evaluable in this study (table 2). At 6 weeks, this number was 90%. Considering the obstacles inherent to research among palliative patients, these are encouraging results. This study also demonstrates that patients receiving palliative radiation are willing and able to answer selected existential questions regarding their illness and outlook on life.

The survival statistics in table 2 as well as the baseline questionnaire responses with regard to symptom burden and QOL (table 3) demonstrate that many of the patients in this small cohort were in relatively good health, considering their diagnoses of incurable cancer. Although this was not an inclusion criterion, it does potentially limit the generalizability of this pilot study.

Our study included all-comers scheduled to receive fractionated PPR. Treatments were prescribed based on patients' general health and estimated life-expectancies. The group of ten prostate cancer patients who received 50-60 Gy (five or six weeks of treatment) was a subgroup of patients with relatively long life-expectancies (often over a year). In contrast, some of our patients had life expectancies of little more than 12 weeks and were chosen for shorter treatment courses (20-30 Gy), for precisely that reason. Such inhomogeneity of the patient cohort, with respect to life expectancies, may represent a problem in a scientific study, but is a common experience in the palliative cancer care practice.

There is no clear consensus for the optimal dose or schedule of PPR in prostate, rectal and bladder cancers. Preferred radiation dose and method of delivery often depend not only on target symptom and tumor type, but also on a range of non-clinical factors such as distance to treatment center. Just among the 22 patients studied here, six different fractionation schedules were used, varying in faction sizes from two to four Gy and total doses of 20-60 Gy. These treatment approaches entail significantly different burdens on the patients. A more homogeneous population and fractionation schedule would therefore be needed in order to reach conclusions about the effects and side-effects of the studied treatment.

## Conclusions

This evaluation of symptoms, QOL and existential questions among PPR patients at 6 and 12 weeks after treatment yields encouraging response rates. The greatest challenge is patient withdrawal because of clinical deterioration. While it is inherent in the population we are studying, this problem is beyond the scope of the protocol and must therefore be taken into consideration in further protocol development. The availability of a radiation therapist to assist patients during data collection appears to have contributed to response rates. The procedure used among these 21 heterogeneous study patients has shown feasibility and is therefore being implemented in a larger Norwegian multicenter study with a more uniform treatment regimen and sample of prostate and rectal cancer patients, in order to reach clinically significant conclusions about the effects of PPR [28].

## Competing interests

The authors declare that they have no competing interests.

## Authors' contributions

MC, IV, CK, RvH, GR and SDF contributed to the development of the idea for this pilot study. MC, RvH and CK administered the study and collected data. MC and GR entered the data and together with IV, analyzed the data. MC wrote the first draft. IV, CK, RvH, GR and SDF provided feedback on the manuscript and all six authors approved the final version.
